# Clinical Diagnosis Codes Identify Patients Unlikely to Receive Orders for Fecal Immunochemical Tests

**DOI:** 10.1177/10732748261465116

**Published:** 2026-06-30

**Authors:** Andrew Tong Li, Shohei Burns, Dalia Martinez, Patricia Wang, Sona Aggarwal, Michael B. Potter, Urmimala Sarkar, Ma Somsouk

**Affiliations:** 1 School of Medicine, University of California, San Francisco, San Francisco, CA, USA; 2 Department of Medicine, University of California, San Francisco, San Francisco, CA, USA; 3 Academic Research Services, University of California, San Francisco, San Francisco, CA, USA; 4 Department of Public Health, 584054 San Francisco Health Network, San Francisco, CA, USA; 5 Department of Family and Community Medicine, University of California, San Francisco, San Francisco, CA, USA; 6 Division of General Internal Medicine, Zuckerberg San Francisco General Hospital and Trauma Center, San Francisco, CA, USA; 7 Division of Gastroenterology, Zuckerberg San Francisco General Hospital and Trauma Center, San Francisco, CA, USA

**Keywords:** colorectal cancer prevention, panel management, population health, organized outreach, high-value care, disparities

## Abstract

**Introduction:**

Automated colorectal cancer (CRC) screening programs often invite all age-eligible patients for fecal immunochemical testing (FIT), but providers may defer screening in clinically complex patients. However, such nuanced decisions are not captured by most automated systems. We evaluated whether International Classification of Diseases, Tenth Revision (ICD-10) codes are associated with variations in FIT ordering patterns beyond age-based outreach criteria.

**Methods:**

In a retrospective observational study of 15,020 screening-eligible patients aged 50-75, we compared the frequency of ICD-10 codes between patients with and without a FIT order. We repeated the analysis restricted to those with a Charlson Comorbidity Index (CCI)<5.

**Results:**

Overall, 4,833 (32.2%) patients did not have a FIT order. Of the 1,215 ICD-10 codes examined, 96 were significantly associated with the absence of a FIT order (p<0.05). These codes frequently indicated digestive diseases (e.g., colonic neoplasm, diverticular disease), advanced comorbidities (e.g., frailty, paralysis), and acute conditions (e.g., fractures, severe infections). Among the patients with CCI<5, 41 codes were associated with the absence of a FIT order (p<0.05), such as heart failure and chronic kidney disease.

**Conclusion:**

Screening-eligible patients without a FIT order were more likely to have ICD-10 codes reflecting digestive diseases, chronic comorbidities, and acute conditions. These findings demonstrate that specific diagnostic profiles are associated with real-world FIT ordering patterns, highlighting the need for further investigation into how comorbidities and competing clinical priorities relate to CRC screening decisions.

## Study Highlights

WHAT IS KNOWN

• Colorectal cancer screening outreach is often based on eligibility and age.
• Primary care providers may defer fecal immunochemical test (FIT) screening for clinically complex patients due to coexisting conditions.

WHAT IS NEW HERE

• Patients eligible for screening but without a FIT order share the same conditions.
• These conditions appear largely to be digestive diseases, chronic comorbidities, and acute conditions.
• Those with a low Charlson Comorbidity Index tended to have chronic comorbidities such as heart failure and chronic kidney disease.

## Introduction

Colorectal cancer (CRC) screening has effectively reduced CRC incidence and mortality.^1^ To increase screening rates in underserved populations, safety-net health systems increasingly rely on the fecal immunochemical test (FIT) for organized outreach due to its low cost, noninvasive nature, high adherence, and ease of use compared to other CRC screening modalities, including colonoscopy and multitarget stool DNA testing (e.g., Cologuard).^2,3^ Because FIT is the most accessible modality for initiating screening, FIT ordering serves as a practical measure of real-world screening activity.^4^

United States Preventive Services Task Force (USPSTF) guidelines recommend selectively offering CRC screening to adults aged 76–85 years with significant comorbidities or limited life expectancy,^5^ reflecting the approximately 10-year lag time to mortality benefit.^6^ In patients with life-limiting illnesses, the potential burden of screening may outweigh long-term benefit.^7^ Consistent with this, providers often prioritize acute health concerns,^8^ and screening is less likely among patients with greater comorbidity or abnormal laboratory results.^9^ These patterns suggest that FIT screening may be selectively deferred in clinically complex patients. However, whether similar selective screening occurs among screening-eligible adults aged 45–75 years remains unclear.

While providers make these nuanced screening decisions in clinical practice, automated systems are not designed to capture them. Screening variation is often attributed to easily measurable variables such as age or family history,^10,11^ whereas a patient’s broader health status is distributed across numerous diagnostic codes. The volume and complexity of International Classification of Diseases, Tenth Revision (ICD-10) codes pose challenges for registry-based approaches,^12,13^ potentially obscuring factors associated with the absence of screening orders. Accordingly, many organized outreach programs do not formally incorporate ICD-10 codes to defer CRC screening in patients with significant comorbidity.^14^

We hypothesized that ICD-10 codes could identify both life-limiting conditions and acute illnesses associated with FIT ordering. The objective was to assess whether specific diagnoses were associated with variations in FIT ordering patterns. Identifying these codes may help characterize clinical contexts associated with FIT ordering patterns and inform future studies evaluating the relationship between comorbidities and CRC screening behavior.

## Methods

### Study Design and Reporting

This is a retrospective observational study using routinely collected electronic health record (EHR) data. The reporting of this study conforms to the RECORD (Reporting of studies Conducted using Observational Routinely-collected Health Data) statement.^15^

### Cohort Development and Definition

**Figure 1.**
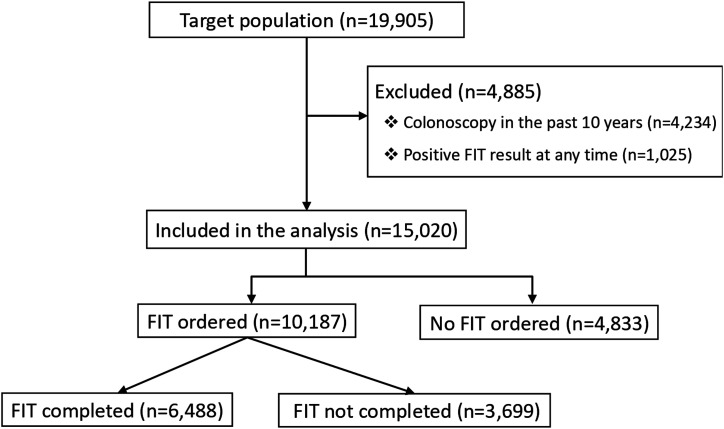
Flow Diagram of the patients eligible for CRC screening with a primary care encounter. Patients included in the analysis were divided by the occurrence of a FIT order

We included screen-eligible patients ages 50 to 75 in a safety-net health system (San Francisco Health Network) with at least one primary care provider (PCP) encounter from June 26, 2022 to June 26, 2023 (Figure 1). During the study period, screening eligibility was based on age between 50 and 75. Although the recommended age to begin CRC screening was lowered from 50 to 45 in May 2021, the EHR had not yet been updated to include care gap alerts for patients age 45 to 49. Patients were selected consecutively from the EHR based on the predefined inclusion criteria of being screen-eligible and having at least one PCP encounter within the study period. We excluded patients from the analytic cohort who either had a documented colonoscopy in the past 10 years or ever had a positive FIT result at any time, since these patients would also be ineligible for FIT screening. Screen-eligible patients were categorized as either having a FIT ordered or no FIT ordered between June 26, 2022, and June 26, 2023. We chose to conduct our study within this safety-net health system because FIT is routinely offered for CRC screening, while colonoscopy can be selected at the discretion of either the PCP or the patient. Since colonoscopy is performed for both screening and diagnostic indications, and the goal of the analysis is to understand FIT ordering patterns, we did not use colonoscopy to define the cohorts.

### Covariates and Outcome Variable

In addition to patient demographic data, we extracted all orders for FIT and all unique ICD-10 codes. The codes were not limited to those “linked” to a specific FIT order or encounter; rather, they represent the patient’s comprehensive diagnostic profile. FIT orders were identified by extracting internal clinical order records from the EHR using unique order identifiers and timestamps. We analyzed “ordered” status as the primary outcome to capture the point-of-care clinical workflow prior to laboratory processing or billing. Current Procedural Terminology (CPT) billing codes were not utilized for identification, as FIT order entry captures the initial clinical workflow point-of-care entry prior to laboratory processing or billing.

We also extracted the Charlson Comorbidity Index (CCI) score directly from the EHR. The CCI is a validated method of classifying prognostic comorbidity with a weighted score (range 0–29) assigned based on the presence of 17 chronic conditions, where higher scores indicate a greater burden of chronic disease.^16^ In clinical validation studies, the CCI has demonstrated high discriminative ability for mortality and healthcare utilization, typically yielding C-statistics between 0.80 and 0.90.^17^ We included the CCI in our analysis to account for overall health status aside from the individual ICD-10 codes.

To ensure sufficient statistical power and clinical relevance, ICD-10 codes were aggregated at the three-character category level by truncating decimal-level specificity. This approach allowed for a robust comparison of disease categories (e.g., K57 for Diverticular Disease) across the study population while minimizing the impact of sparse data at the sub-code level.

To ensure internal consistency, we adopted the term “comorbidities” to describe the full spectrum of acute and chronic clinical conditions identified via ICD-10 diagnostic codes. We define these comorbidities as any concurrent medical diagnoses present in the EHR at the time of screening eligibility, which allow us to evaluate their statistical association with FIT ordering rates. The comorbidities were categorized based on two frameworks. First, they were categorized into both chronic comorbidities (e.g., end-stage organ failure, advanced malignancy) and acute comorbidities (e.g., transient illnesses such as infections, acute pain). Second, they were categorized based on organ system or disease type (e.g., cardiovascular, digestive, neoplasm).

### Data Retrieval and Security

Patient data, including medical record numbers (MRNs), were extracted from the EHR by the clinical informatics specialist. To maintain the security of Protected Health Information, all identifiable data were stored and analyzed strictly within the UCSF Research Analysis Environment. This secure, HIPAA-compliant platform ensures that all identifiable elements remain within a controlled, audited environment accessible only to IRB-approved investigators. All patient details were de-identified for the final analysis to ensure that the identity of any person could not be ascertained.

### Analysis

We summarized patient demographic characteristics for the two cohorts, “Patients with FIT ordered” and “Patients with no FIT ordered,” using proportions and means as appropriate. The primary analysis enumerated and compared the frequency of each ICD-10 code between the two cohorts using the odds ratios (OR) along with the corresponding 95% confidence interval (CI) using the Fisher’s exact test with Benjamini-Hochberg correction for multiple hypothesis testing.^18^ Given the exploratory nature of this study, we focused our attention beyond statistical significance and onto substantially low odds ratios with values below a threshold of 0.6, which were presumed to be more clinically relevant.

To isolate barriers to screening that are independent of limited life expectancy, a secondary sensitivity analysis was performed on a restricted cohort of low comorbidity patients with a CCI < 5.^19^ Given that USPSTF screening guidelines suggest a reduced benefit for individuals with significant multimorbidity and limited longevity, this sub-analysis allowed us to determine if specific ICD-10 codes remain associated with the absence of a FIT screening order even among patients who are otherwise candidates for long-term preventive care.

To validate the sensitivity of our analyses, we intentionally included ICD-10 codes for conditions that preclude average-risk colorectal cancer screening per USPSTF guidelines.^5^ These included prior diagnoses of colorectal cancer, genetic predisposition to cancer, inflammatory bowel disease, and conditions with symptomatic rectal bleeding such as hemorrhoids and perianal venous thrombosis. These variables served as internal validation controls. Similarly, codes for symptomatic conditions like diverticular disease were included to assess the system’s ability to capture clinical scenarios where diagnostic colonoscopy is preferred over FIT screening. We hypothesized that if our statistical approach accurately reflects clinical appropriateness, these “established” exclusion criteria would demonstrate a strong and significant association with the absence of a FIT order. This would provide a baseline for evaluating the clinical relevance of other, less-traditional comorbidities identified in our dataset.

All statistical analyses, figures, and tables were completed with Microsoft Excel (version 16), RStudio (version 4.3.1), and Stata MP 18.

### Ethical Considerations and Informed Consent

This study was reviewed and approved by the University of California, San Francisco Institutional Review Board (IRB #13-11900). A waiver of informed consent and HIPAA authorization was granted for this retrospective analysis, as the research involved no more than minimal risk to subjects and utilized a secure, institutional computing environment to protect patient identifiers, including MRNs. All study procedures were performed in accordance with the ethical standards of the 1964 Declaration of Helsinki and its later amendments.

## Results

### Patient Demographics

There were 19,905 patients aged 50-75 in the health network with a primary care encounter during the study period, of whom 15,020 were eligible for CRC screening. Among eligible patients, 10,187 (67.8%) had a FIT order during the 12-month study period (FIT-ordered group), while 4,833 (32.2%) did not (No FIT ordered group) (Table 1). Among patients in the FIT-ordered group, 6,488 (63.7%) completed their FIT.

**Table 1.** Demographic Characteristics for Patients Eligible for CRC Screening and the Proportion of Patients With and Without an Order for FIT

Demographic characteristics	Total	Patients with FIT ordered	Patients with no FIT ordered	P-value
Patient Count	15,020 (100%)	10,187 (100%)	4,833 (100%)	​
**Sex**, n (%)	​	​	​	<0.001
Female	7,632 (50.8%)	5,385 (52.9%)	2,247 (46.5%)	​
Male	7,379 (49.1%)	4,797 (47.1%)	2,582 (53.4%)	​
Other/Unknown	9 (0.1%)	5 (0%)	4 (0.1%)	​
**Age**, n (%)	​	​	​	<0.001
50-54	2,965 (19.7%)	2,209 (21.7%)	756 (15.6%)	​
55-59	3,369 (22.4%)	2,293 (22.5%)	1,076 (22.3%)	​
60-64	3,611 (24%)	2,452 (24.1%)	1,159 (24%)	​
65-69	2,889 (19.2%)	1,879 (18.4%)	1,010 (20.9%)	​
70-75	2,186 (14.6%)	1,354 (13.3%)	832 (17.2%)	​
**Race**, n (%)	​	​	​	<0.001
Asian	5,121 (34.1%)	3,686 (36.2%)	1,435 (29.7%)	​
Black Or African American	1,988 (13.2%)	1,225 (12%)	763 (15.8%)	​
Hispanic or Latino	4,220 (28.1%)	2,920 (28.7%)	1,300 (26.9%)	​
Other/Unknown	845 (5.6%)	576 (5.7%)	269 (5.6%)	​
White	2,846 (18.9%)	1,780 (17.5%)	1,066 (22.1%)	​
**Language**, n (%)	​	​	​	<0.001
Chinese	3,358 (22.4%)	2,502 (24.6%)	856 (17.7%)	​
English	7,189 (47.9%)	4,495 (44.1%)	2,694 (55.7%)	​
Other/Unknown	1,260 (8.4%)	894 (8.8%)	366 (7.6%)	​
Spanish	3,213 (21.4%)	2,296 (22.5%)	917 (19%)	​
**Insurance**, n (%)	​	​	​	<0.001
County Sponsored	552 (3.7%)	428 (4.2%)	124 (2.6%)	​
Healthy Worker	3,389 (22.6%)	2,478 (24.3%)	911 (18.8%)	​
Medicaid	7,347 (48.9%)	5,038 (49.5%)	2,309 (47.8%)	​
Medicare	3,534 (23.5%)	2,118 (20.8%)	1,416 (29.3%)	​
Other	198 (1.3%)	125 (1.2%)	73 (1.5%)	​
**Marital Status**, n (%)	​	​	​	<0.001
Divorced	1,378 (9.2%)	898 (8.8%)	480 (9.9%)	​
Married	5,253 (35%)	3,779 (37.1%)	1,474 (30.5%)	​
Other/Unknown	135 (0.9%)	99 (1%)	36 (0.7%)	​
Separated	572 (3.8%)	379 (3.7%)	193 (4%)	​
Single	6,908 (46%)	4,512 (44.3%)	2,396 (49.6%)	​
Widowed	774 (5.2%)	520 (5.1%)	254 (5.3%)	​
**Homeless**, n (%)	​	​	​	<0.001
Yes	2,245 (14.9%)	1,340 (13.2%)	905 (18.7%)	​
No	12,775 (85.1%)	8,847 (86.8%)	3,928 (81.3%)	​

*Note.* P-values were calculated using Pearson’s chi-square test.

Compared with patients who received a FIT order, those without a FIT order were more likely to be older, African American, English-speaking, Medicare-insured, divorced, and homeless. Overall, Pearson’s chi-square tests demonstrated statistically significant differences across all demographic variables between both groups (all *P* < 0.001). Given the large sample size, some statistically significant differences are modest in magnitude and may not be clinically meaningful.

The study population was representative of an urban safety-net system. Notably, 905 patients (18.7%) were identified as homeless in the structured EHR social history. Additionally, a significant proportion of the study population were enrolled in city-sponsored insurance programs, including County Sponsored (n=124) and Healthy Worker (n=911) plans.

### ICD-10 coding categories associated with orders for FIT

**Figure 2.**
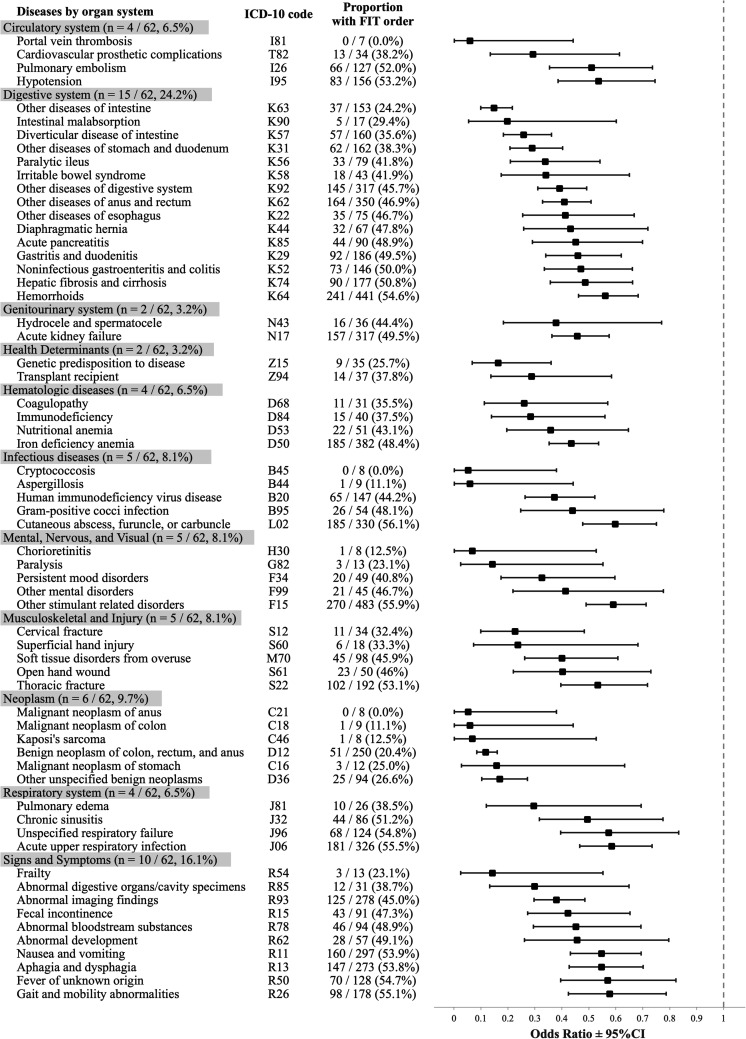
ICD-10 codes with an OR < 0.6 (n = 62), grouped by disease category, that were significantly associated with patients who did not have an order for FIT. Significance was determined by a two-sided Fisher’s exact test (*P* < 0.05) with Benjamini−Hochberg correction for multiple testing. Codes within each category are ordered based on OR from lowest to highest. Note that established USPSTF exclusion criteria (e.g., prior colorectal cancer, genetic predisposition to disease) were included as internal validation controls for sensitivity analysis

We examined 1,215 ICD-10 codes. 96 unique ICD-10 codes were significantly associated with reduced odds of a FIT order (OR < 1). Within this group, 62 codes (60.2%) demonstrated an OR < 0.6, including 25 codes (24.3%) with an OR < 0.3 (Figure 2). Separately, 7 codes were significantly associated with increased odds of a FIT order (OR > 1) (data not shown). Several categories of codes emerged, such as codes related to digestive disease, chronic comorbidities, and acute comorbidities.

Digestive disease codes representing benign and malignant neoplasms of the gastrointestinal tract were strongly associated with an absence of FIT orders, such as malignant neoplasm of the anus (ICD-10 code C21, odds ratio (OR) 0.05, 95% CI [0.001-0.38]), malignant neoplasm of the colon (C18, OR 0.06 [0.001-0.44]), Kaposi’s sarcoma (C46, OR 0.07 [0.002-0.53]), benign neoplasm of the colon, rectum, and anus (D12, OR 0.12 [0.08-0.16]), malignant neoplasm of the stomach (C16, OR 0.16 [0.03-0.63]), and other unspecified benign neoplasms (D36, OR 0.17 [0.10-0.27]). In addition, non-neoplastic conditions of the gastrointestinal tract associated with absence of FIT orders included intestinal malabsorption (K90, OR 0.20 [0.05-0.60]), diverticular disease of the intestine (K57, OR 0.26 [0.18-0.36]), and other diseases of the intestine (K63, OR 0.15 [0.10-0.22]) and of the stomach and duodenum (K31, OR 0.29 [0.21-0.40]).

Comorbidities were also significantly associated with the absence of a FIT order. The most differentially represented ICD-10 codes were frailty (R54, OR 0.14 [0.03-0.55]), paralysis (G82, OR 0.14 [0.03-0.55]), immunodeficiency (D84, OR 0.28 [0.14-0.56]), genetic predisposition to disease (Z15, OR 0.16 [0.07-0.36]), transplant recipient (Z94, OR 0.29 [0.14-0.58]), coagulopathy (D68, OR 0.26 [0.11-0.57]), portal vein thrombosis (I81, OR 0.06 [0.001-0.44]), and cardiovascular prosthetic complications (T82, OR 0.29 [0.13-0.61]). Other clinical conditions with an OR > 0.3 included chronic obstructive pulmonary disease (J44, OR 0.72 [0.59-0.88]), chronic kidney disease (N18, OR 0.66 [0.56-0.78]), human immunodeficiency virus disease (B20, OR 0.37 [0.26-0.52]), and heart failure (I50, OR 0.66 [0.56-0.78]).

We found that several acute clinical conditions were also associated with an absence of a FIT order. The most discriminating ICD-10 codes with an OR < 0.3 included cervical fracture (S12, OR 0.23 [0.10-0.48]), superficial hand injury (S60, OR 0.24 [0.07-0.68]), chorioretinitis (H30, OR 0.07 [0.002-0.53], and infections such as cryptococcosis (B45, OR 0.05 [0.001-0.38]) and aspergillosis (B44, OR 0.06 [0.001-0.44]).

Of note, there were several codes associated with an elevated likelihood of having a FIT order; these included malignant neoplasm of thyroid gland (C73, OR 10.93 [1.77-449.68]), abnormal specimens from female genital organs (R87, OR 1.54 [1.17-2.04]), synovitis and tenosynovitis (M65, OR 1.37 [1.11-1.7]), abnormal blood-pressure reading (R03, OR 1.27 [1.08-1.49]), disorders of lipoprotein metabolism and other dyslipidemias (E78, OR 1.25 [1.14-1.37]), essential hypertension (I10, OR 1.17 [1.06-1.29]), and elevated blood glucose level (R73, OR 1.17 [1.07-1.29]).

**Figure 3.**
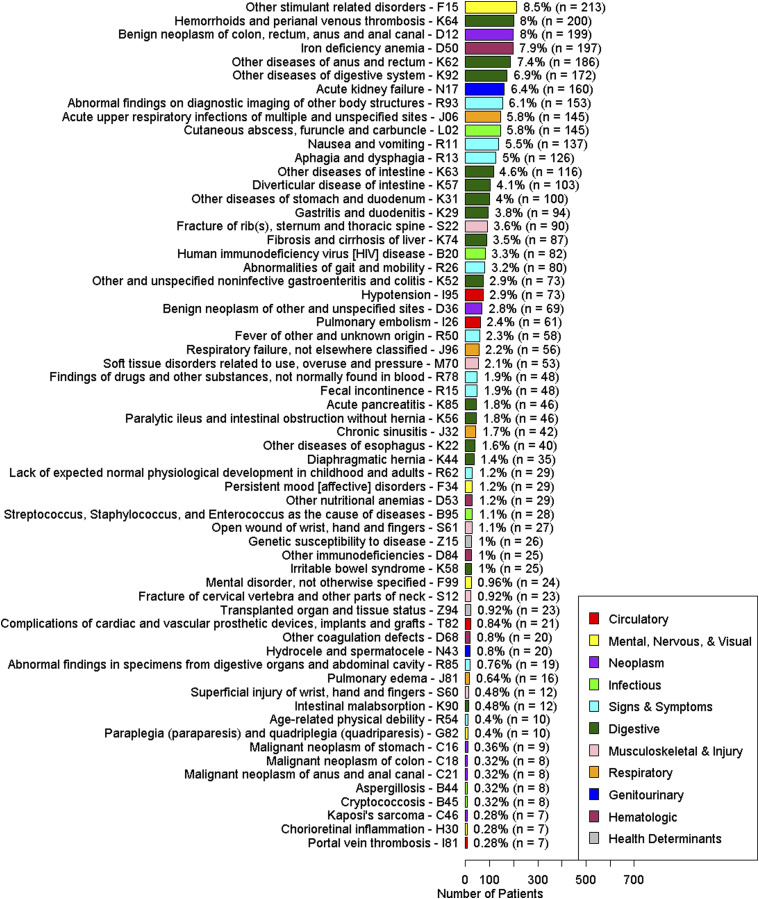
Count and proportion of patients in the cohort with no FIT ordered according to each ICD-10 code

Of the 62 ICD-10 codes with OR < 0.6, the most prevalent conditions among the 4,833 patients with no FIT ordered were other stimulant related disorders (F15, OR 0.59 [0.49-0.71]), hemorrhoids and perianal venous thrombosis (K64, OR 0.56 [0.46-0.68]), benign neoplasm of colon, rectum, and anus (D12, OR 0.12 [0.08-0.16]), iron deficiency anemia (D50, OR 0.44 [0.35-0.54]), and other diseases of anus and rectum (K62, OR 0.41 [0.33-0.51]) (Figure 3).

### ICD-10 coding categories in patients with low CCI

We found that patients with increasing comorbidities according to the CCI were significantly less likely to have a FIT order: 74.0% (3609/4880) for CCI 0, 72.2% (2501/3463) for CCI 1, 68.5%(1181/1723) for CCI 2, 65.0% (884/1361) for CCI 3, 64.3% (506/787) for CCI 4, and 53.7% (1506/2806) for CCI 5+ (p < 0.001) (Supplemental Figure 1). After restricting the patient population into CCI < 5 (n = 12,214), there were 8,681 (71.1%) patients who had an order for FIT and 3,533 (28.9%) without an order for FIT.

There were 41 ICD-10 codes that were significantly associated with reduced odds of FIT order. There were several codes that were new or became more prominent in this analysis; they included comorbidities such as cachexia (R64, OR 0.09 [0.01-0.44]), heart failure (I50, OR 0.55 [0.43-0.71]), chronic kidney disease (N18, OR 0.58 [0.43-0.77]), osteomyelitis (M86, OR 0.42 [0.24-0.73]), and housing and economic circumstances (Z59, OR 0.58 [0.49-0.68]). The remaining codes are found in the Supplemental file.

## Discussion

In this large, diverse population representative of an urban safety-net health system, we found that despite an encounter with the primary care clinic, over 30% of patients who were not up-to-date with CRC screening did not receive an order for a FIT test. The absence of a FIT order was significantly associated with specific categories of clinical diagnoses and disorders, as represented by ICD-10 codes. These findings suggest that ICD-10 diagnostic patterns may be relevant to future investigations on how automated CRC screening programs account for real-time clinical complexity.^14^

As expected, our analysis identified several internal validation controls. The association of prior colorectal cancer, inflammatory bowel disease such as ulcerative colitis and Crohn’s, and hemorrhoids and perianal venous thrombosis with the absence of a FIT order supports the ability of our analysis to detect diagnoses plausibly associated with altered CRC screening practices. We found that patients with other digestive diseases and related conditions were also less likely to receive a FIT order. In these instances, direct visualization with colonoscopy would be prioritized over stool-based testing, since FIT provides no additional diagnostic utility for a patient previously diagnosed with a gastrointestinal cancer.^20^

Comorbidities were also associated with the absence of a FIT order, such as frailty, paralysis, cirrhosis, kidney disease, obstructive pulmonary disease, heart failure, dementia, acquired immunodeficiency syndrome (AIDS), and genetic susceptibility to disease. This is consistent with previous studies which demonstrated a lower uptake of CRC screening in patients with comorbidities,^21-23^ as well as the practice of excluding patients with limited life expectancy from screening.^16,24-26^ In contrast, stable chronic conditions, such as dyslipidemia, hyperglycemia, and hypertension, were associated with higher rates of FIT ordering. While these conditions carry significant long-term morbidity, their presence in this cohort likely represents established longitudinal primary care engagement. Unlike acute injuries or advanced end-organ disease, these manageable chronic conditions may provide more frequent opportunities for preventive health maintenance and routine screening within the primary care workflow, consistent with previous evidence.^22,24^

Less intuitively, we found that patients who harbored ICD-10 codes representing acute illnesses were less likely to have an order for FIT. These conditions were related to traumatic injury, inflammation, infections, and other acute illnesses, some of which have previously been described.^22^ We additionally found that acute illnesses of the blood and circulatory system were associated with the absence of a FIT order, including portal vein thrombosis and surgical complications. Future research is required to determine whether these associations are driven by competing clinical priorities during primary care encounters.

In the population with fewer comorbidities as defined by CCI < 5, we continued to find codes for comorbidities such as heart failure and chronic kidney disease. Additional codes associated with the absence of a FIT order that were not significant in the initial analysis included housing and financial problems, cachexia, and osteomyelitis. This CCI restricted subgroup analysis suggests that factors beyond life expectancy alone prompt deferral. To date, no studies have published results from similar analyses that were restricted to patients with low comorbidity status defined as CCI < 5.

Our results demonstrate distinct variations in FIT ordering rates across different clinical categories. Patients with advanced comorbidities and active health conditions such as musculoskeletal fractures were less likely to have a FIT ordered. Although current guidelines discourage patients with limited life expectancy from getting screened, life expectancy alone does not fully account for the observed variations in FIT screening orders. It may be the case that acute medical demands, such as rehabilitation following a traumatic injury, temporarily shift the focus of primary care encounters, meaning that FIT screening is not addressed until the patient’s health has stabilized.^8,27^

Rather than mandating exclusion from FIT screening, these codes may help characterize clinically complex patient populations for whom CRC screening decisions are more likely to involve individualized clinical consideration. Importantly, this study does not establish that patients with these ICD-10 codes should forgo CRC screening, nor does it evaluate the clinical appropriateness of individual screening decisions. Rather, the findings identify associations between diagnostic coding patterns and FIT ordering behavior within routine primary care practice.

The potential implications of these findings remain exploratory. ICD-10 codes may represent measurable markers of clinical complexity associated with FIT ordering patterns in routine practice. Future studies incorporating chart review, clinician and patient perspectives, and prospective evaluation are needed to determine whether specific diagnostic categories may be useful in future CRC screening risk-stratification or outreach frameworks before any incorporation into screening workflows could be considered. Patient and provider input may also help clarify reasons for deferring screening and inform targeted screening approaches.

The external validity of these findings is supported by our study’s unique setting. While conducted within a single health system, this environment was chosen because it routinely utilizes FIT as its primary, population-based screening modality. Unlike many other practice environments where colonoscopy is the predominant modality, our setting allows for a focused analysis of variations in automated FIT-based outreach relative to patient comorbidities, yielding findings that may be relevant to other large safety-net systems where FIT is the standard of care.

In terms of internal validity, we acknowledge that while demographics and social determinants of health (SDOH) may influence screening rates, our analysis focuses specifically on the granular clinical diagnoses captured by ICD-10 codes. We recognize that many clinical conditions may be correlated with SDOH factors. However, ICD-10 data are large and complex, with a highly imbalanced (long-tailed) distribution in which a small number of codes are common and most occur infrequently^12,13^ (Supplemental Figure 2). Hence, a multiple regression model would lack the statistical power to compare these factors head-to-head. Our findings serve as a preliminary step in identifying which specific clinical “signals” are most strongly associated with variations in real-world FIT ordering, which can later be integrated into more complex, multi-factor predictive models. Furthermore, while a missing FIT order may result from various factors, including unintentional omission or competing clinical priorities, our findings demonstrate a consistent association between specific ICD-10 codes and screening deferral. We do not suggest that these conditions are the sole drivers of provider behavior; rather, they represent measurable clinical markers that could be evaluated by future efforts aimed at better understanding real-world screening practices.

There are important limitations to this study. First, there are multiple ICD-10 codes (K63 –other diseases of intestine, D84 – other immunodeficiencies) that encompass a range of diagnoses, and while associated with FIT orders, are in and of themselves non-specific and do not provide sufficient logic as to why FIT orders were deferred. Chart review and provider interviews will help refine our understanding of the provider’s intention to offer or defer screening. Second, while we used Benjamini-Hochberg correction and a clinical threshold (OR < 0.6), the sheer number of ICD-10 codes precluded a multivariable adjusted analysis. Consequently, residual confounding from unadjusted factors (e.g., patient refusals, socioeconomic status, or encounter frequency) and spurious associations remain possible. Future qualitative studies are needed to better interpret these code associations. Third, orders for FIT could have been independently made by medical assistants and nurses even if screening was not intended by the PCP. As such, inadvertent orders for FIT may have occurred among patients with these ICD-10 codes, and if so, the misclassification of FIT orders likely underestimates the true association. We acknowledge that PCPs may not order a FIT test for an array of reasons, such as prior refusals by the patient, unsuccessful attempts to screen, structural constraints of the visit, such as lack of time or nursing support, failure to remember to offer a FIT, and lack of knowledge or familiarity with FIT screening.^11,28^ Moreover, there could be unconscious biases that play into the decision about when and whether a FIT test is appropriate to order, such as having different subjective judgements about the patient’s life expectancy or compliance with screening. Finally, while our safety-net setting provides high external validity for FIT-based systems, findings may vary in practice environments where commercially insured patients or colonoscopy-primary modalities predominate. Despite these limitations, ICD-10 codes are readily available in the electronic medical records and should be explored as a valuable way to improve screening services.

In summary, certain ICD-10-coded conditions were associated with a lower likelihood of FIT ordering among screening-eligible patients in a safety-net health system. These findings demonstrate that clinical data profiles correspond with variations in real-world CRC screening practices beyond age-based eligibility alone. Further work is needed to investigate how patient clinical profiles, preferences, and encounter dynamics relate to variations in organized CRC screening.

## Supplemental Material

Supplemental Material - Clinical Diagnosis Codes Identify Patients Unlikely to Receive Orders for Fecal Immunochemical Tests Supplemental Material for Clinical Diagnosis Codes Identify Patients Unlikely to Receive Orders for Fecal Immunochemical Tests by Andrew Tong Li, Shohei Burns, Dalia Martinez, Patricia Wang, Sona Aggarwal, Michael Potter, Urmimala Sarkar and Ma Somsouk in Cancer Control.

Supplemental Material - Clinical Diagnosis Codes Identify Patients Unlikely to Receive Orders for Fecal Immunochemical Tests Supplemental Material for Clinical Diagnosis Codes Identify Patients Unlikely to Receive Orders for Fecal Immunochemical Tests by Andrew Tong Li, Shohei Burns, Dalia Martinez, Patricia Wang, Sona Aggarwal, Michael Potter, Urmimala Sarkar and Ma Somsouk in Cancer Control.

## Appendix

### Abbreviations

AIDS: Acquired immunodeficiency syndrome
CCI: Charlson Comorbidity Index
CRC: Colorectal cancer
EHR: Electronic health record
FIT: Fecal immunochemical test
ICD-10: International Classification of Disease, Tenth Revision
PCP: Primary care provider
SDOH: Social determinants of health
USPSTF: United States Preventive Services Task Force.
