# Supplementary material for: Clinical Diagnosis Codes Identify Patients Unlikely to Receive Orders for Fecal Immunochemical Tests
Source: Cancer Control. 2026 Jun 30;33:10732748261465116. doi: 10.1177/10732748261465116 (PMC13319795; doi:10.1177/10732748261465116)

Supplementary Figure. 1. Count and proportion of patients in the cohort with FIT ordered according to Charlson Comorbidity Index (CCI).

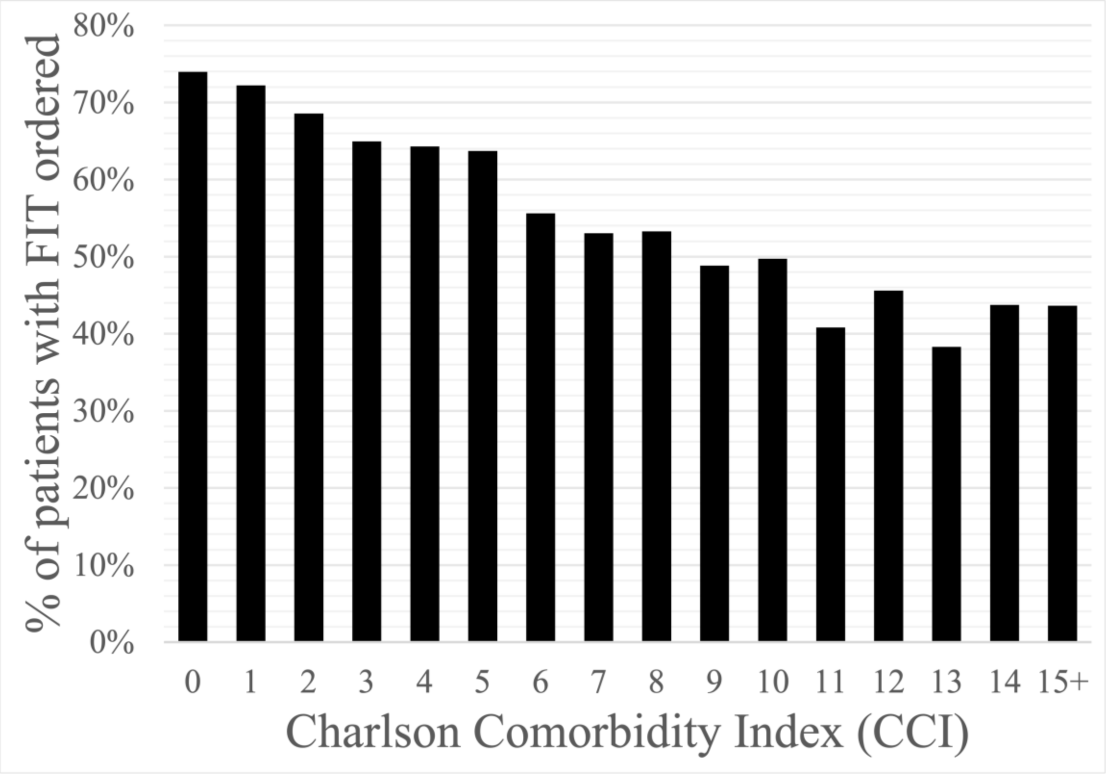

Supplementary Figure 2. ICD-10 codes (n = 1,215) distributed according to their odds ratio (OR) and patient count, plotted on a logarithmic scale. Codes with an OR < 0.6 are represented by the vertical red dashed line. Codes marked with an "x" were not significant (n = 1,112), codes with hollow circles were significant and OR > 0.6 (n = 41), and solid circles were significant and OR < 0.6 (n = 62). Significance was determined to be $P < 0.05$ based on two-sided Fisher's exact tests. Benjamini−Hochberg correction for multiple tests was applied. Rank is the ratio of patient count to odds ratio; high-ranked codes have a low odds ratio and high patient count

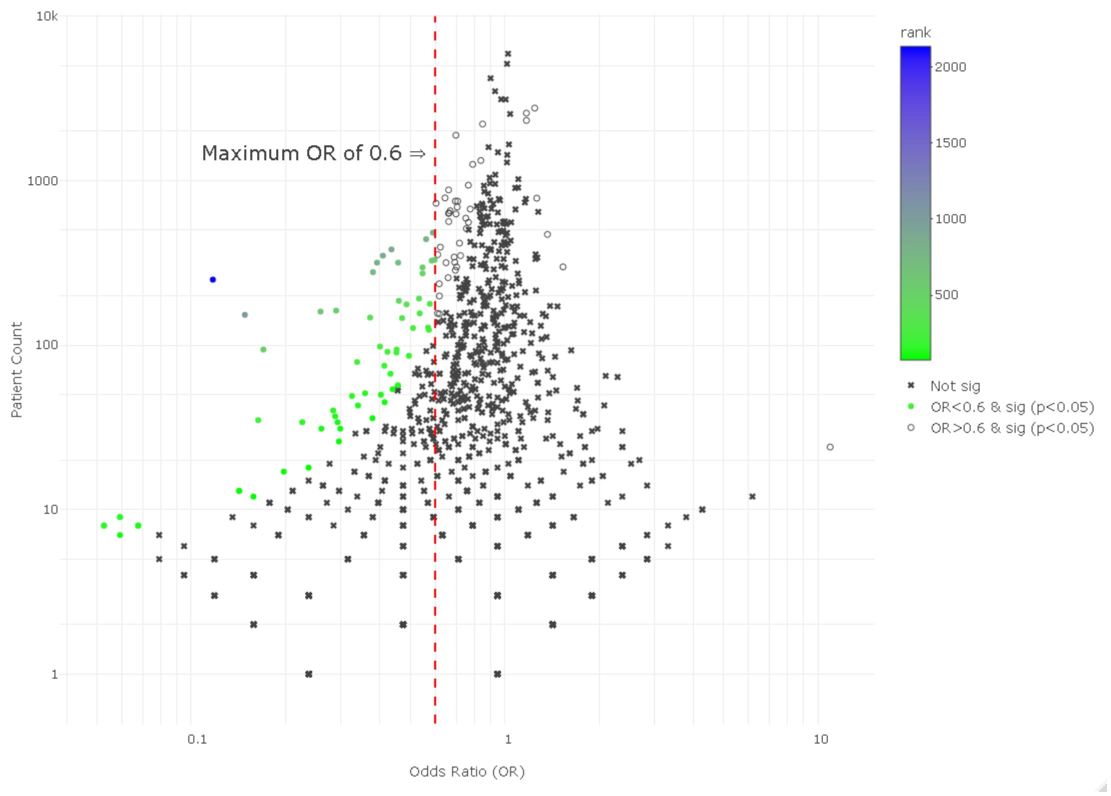

Supplement: Supplemental Material - Clinical Diagnosis Codes Identify Patients Unlikely to Receive Orders for Fecal Immunochemical Tests [file sj-pdf-1-ccx-10.1177_10732748261465116.pdf]
